# Supplementary material for: Evolutionary history of Coleoptera revealed by extensive sampling of genes and species
Source: Nat Commun. 2018 Jan 15;9:205. doi: 10.1038/s41467-017-02644-4 (PMC5768713; doi:10.1038/s41467-017-02644-4)
Supplement: Supplementary file 1 — Supplementary Information [file 41467_2017_2644_MOESM1_ESM.pdf]

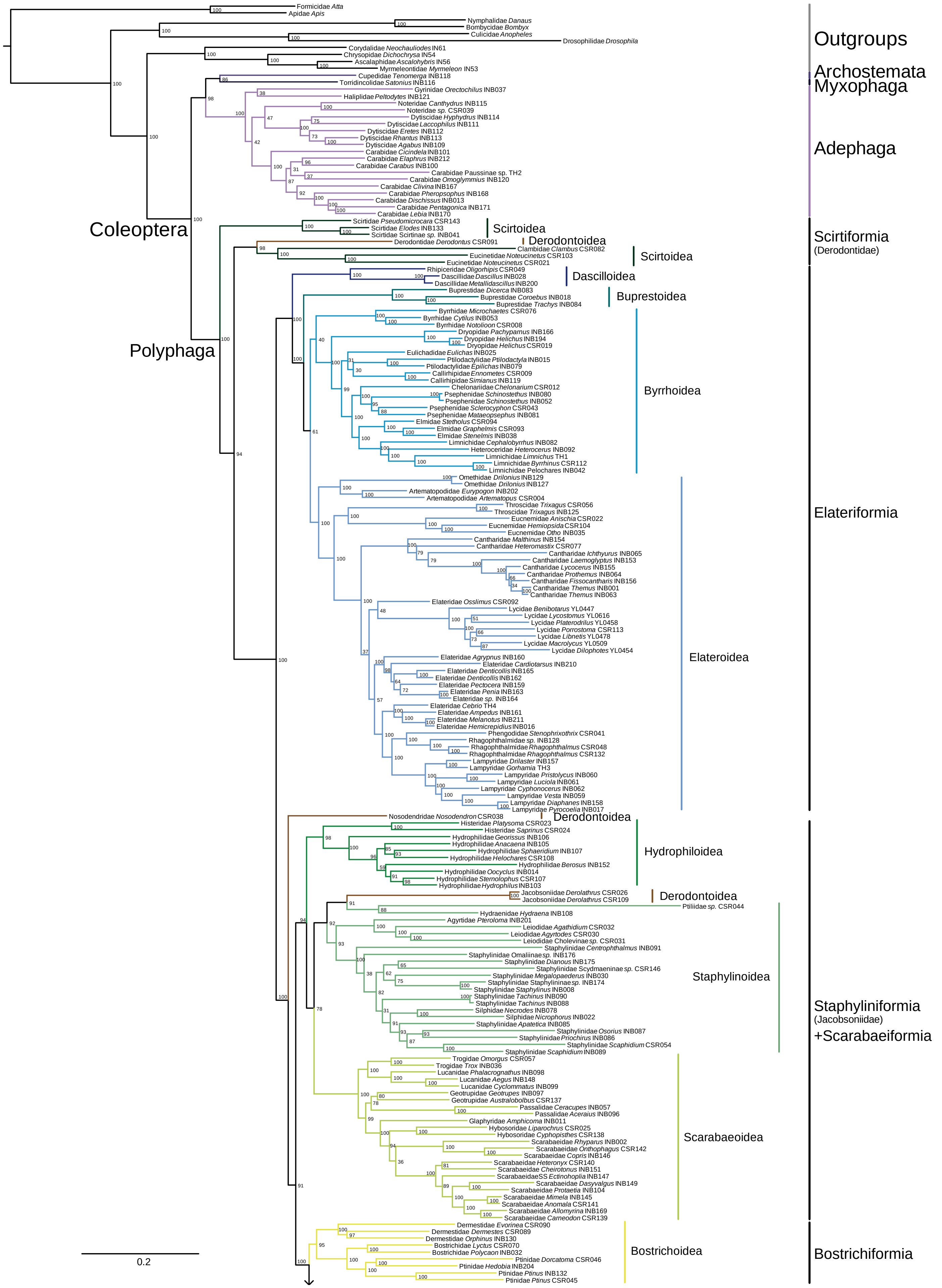

Supplementary Figure 1. The best ML tree inferred by RAxML based on the concatenated amino acid dataset (23,802 AAs) of 383 species with 10 rate-partitions under their best-fit models. Numbers beside nodes are supports after 500 bootstrap replicates. Different clades are colored according to the classification.

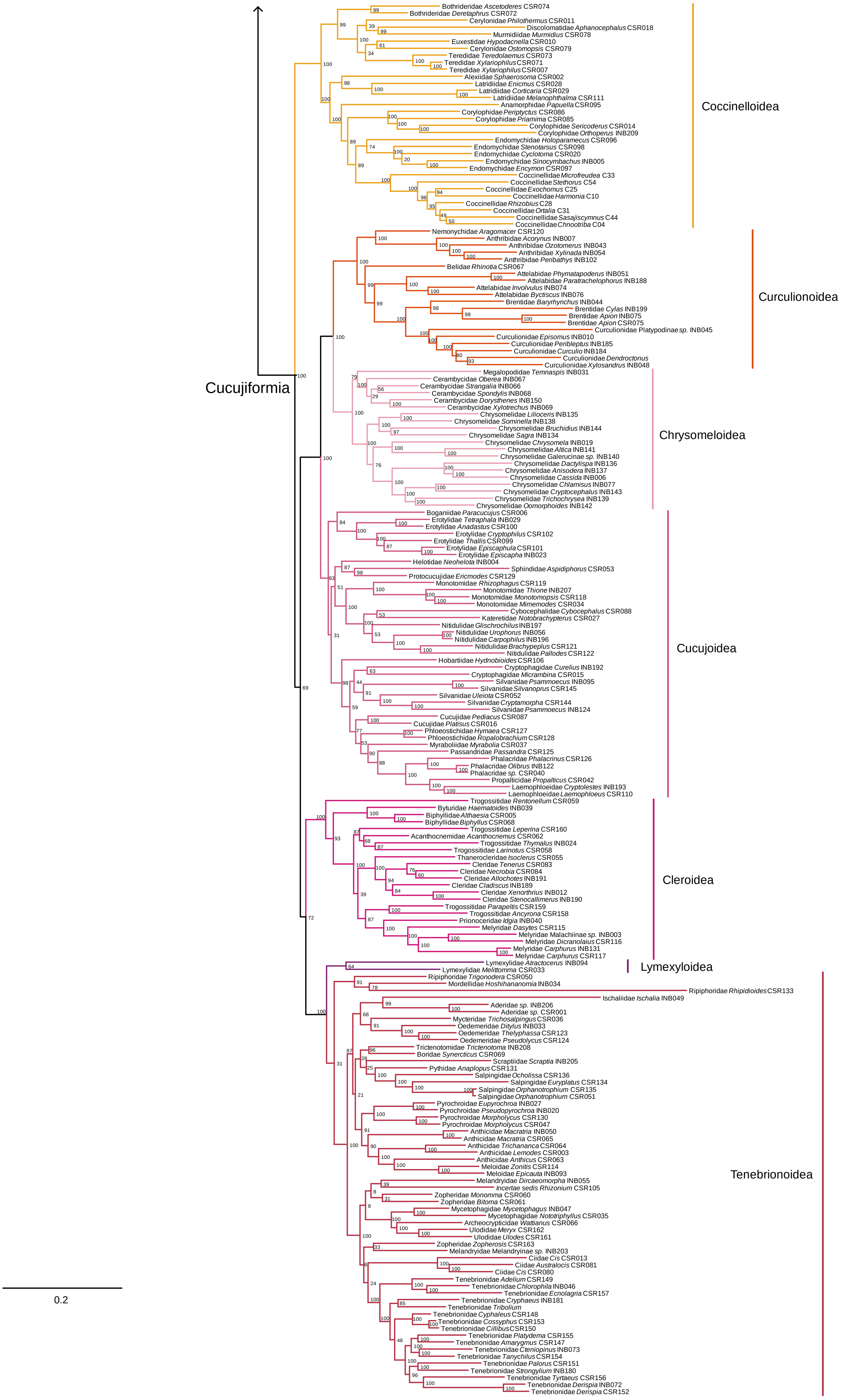

Supplementary Figure 1. (Continued).

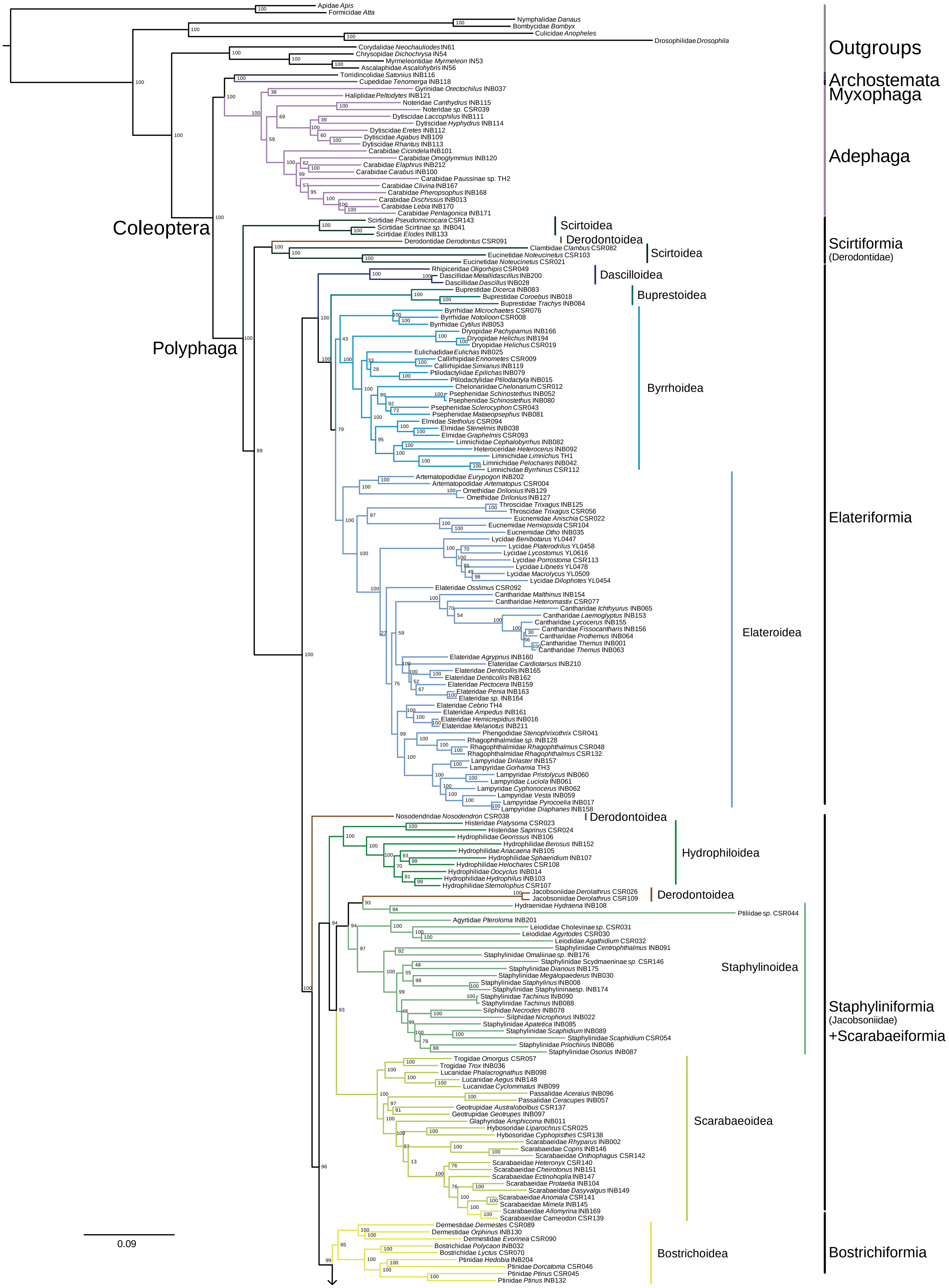

Supplementary Figure 2. The best ML tree inferred by RAxML based on the concatenated degenerated nucleotide matrix (71,406 bp) of 383 species with three codon partitions under the GTR+GAMMA model. Numbers beside nodes are supports after 500 bootstrap replicates. Different clades are colored according to the classification.

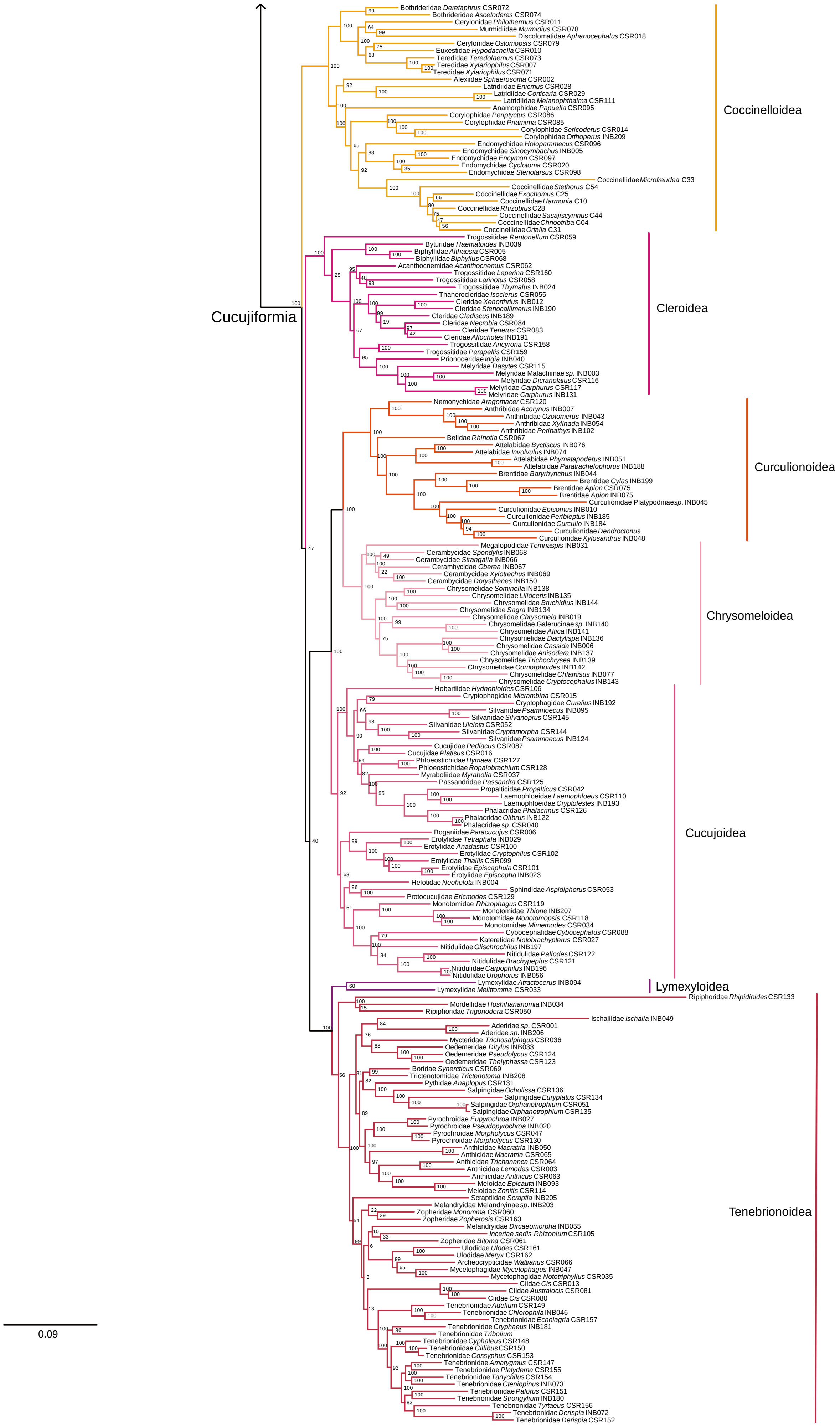

Supplementary Figure 2. (Continued).

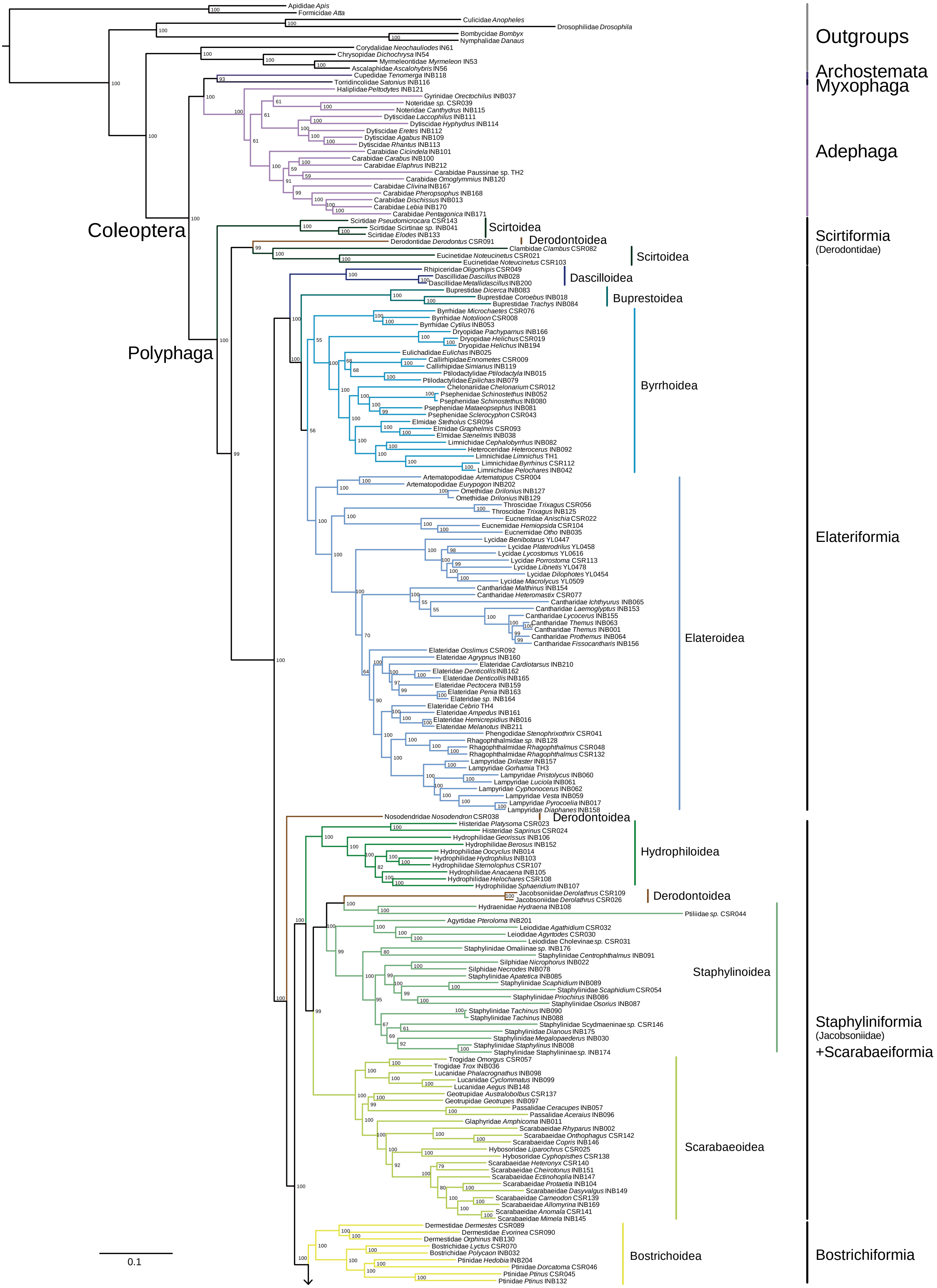

Supplementary Figure 3. The best ML tree inferred by IQ-TREE based on the concatenated amino acid dataset (23,802 AAs) of 383 species with 10 rate-partitions under their best-fit models. Numbers beside nodes are supports values after 5000 ultrafast bootstrap replicates. Different clades are colored according to the classification.

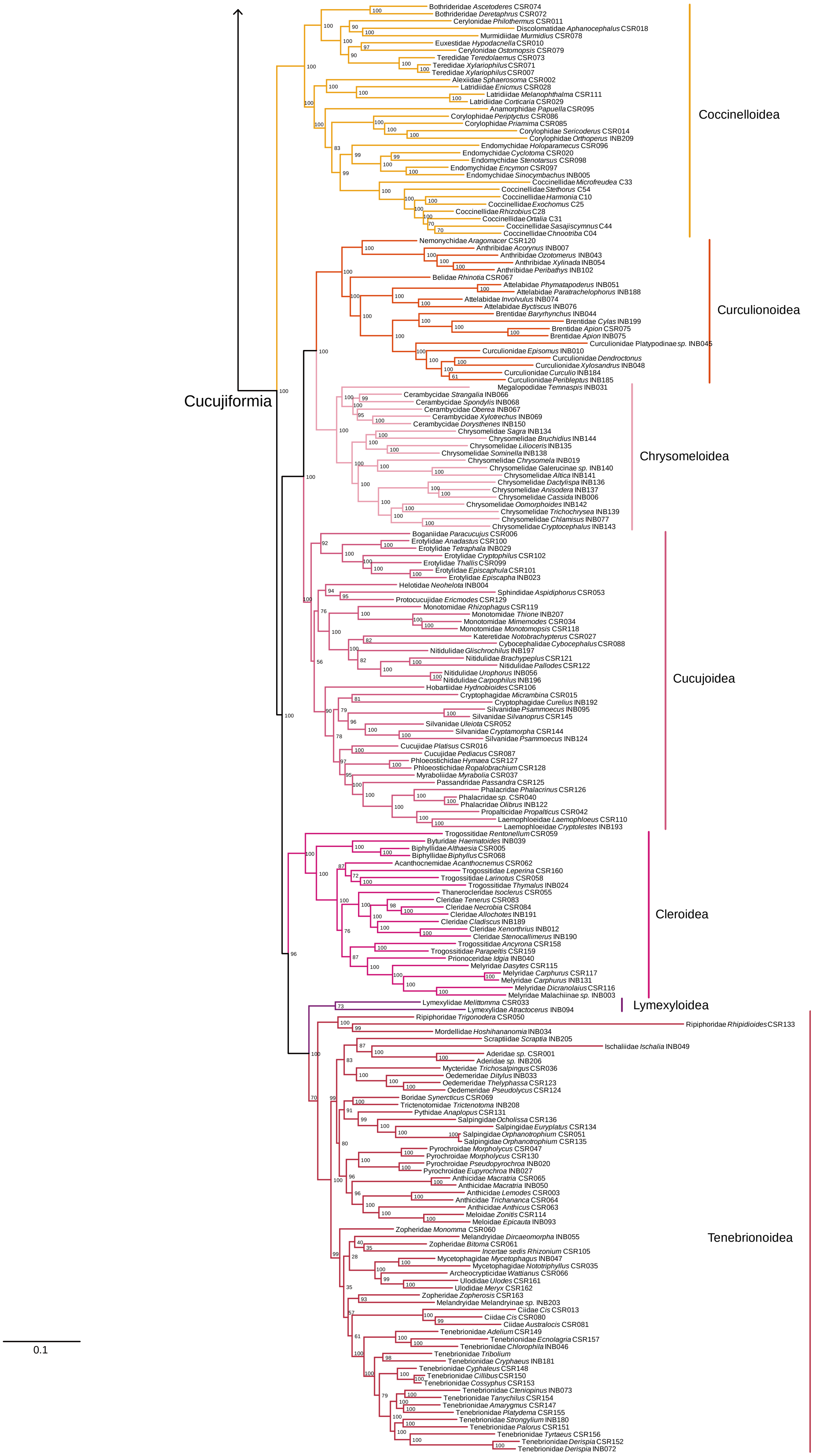

Supplementary Figure 3. (Continued).

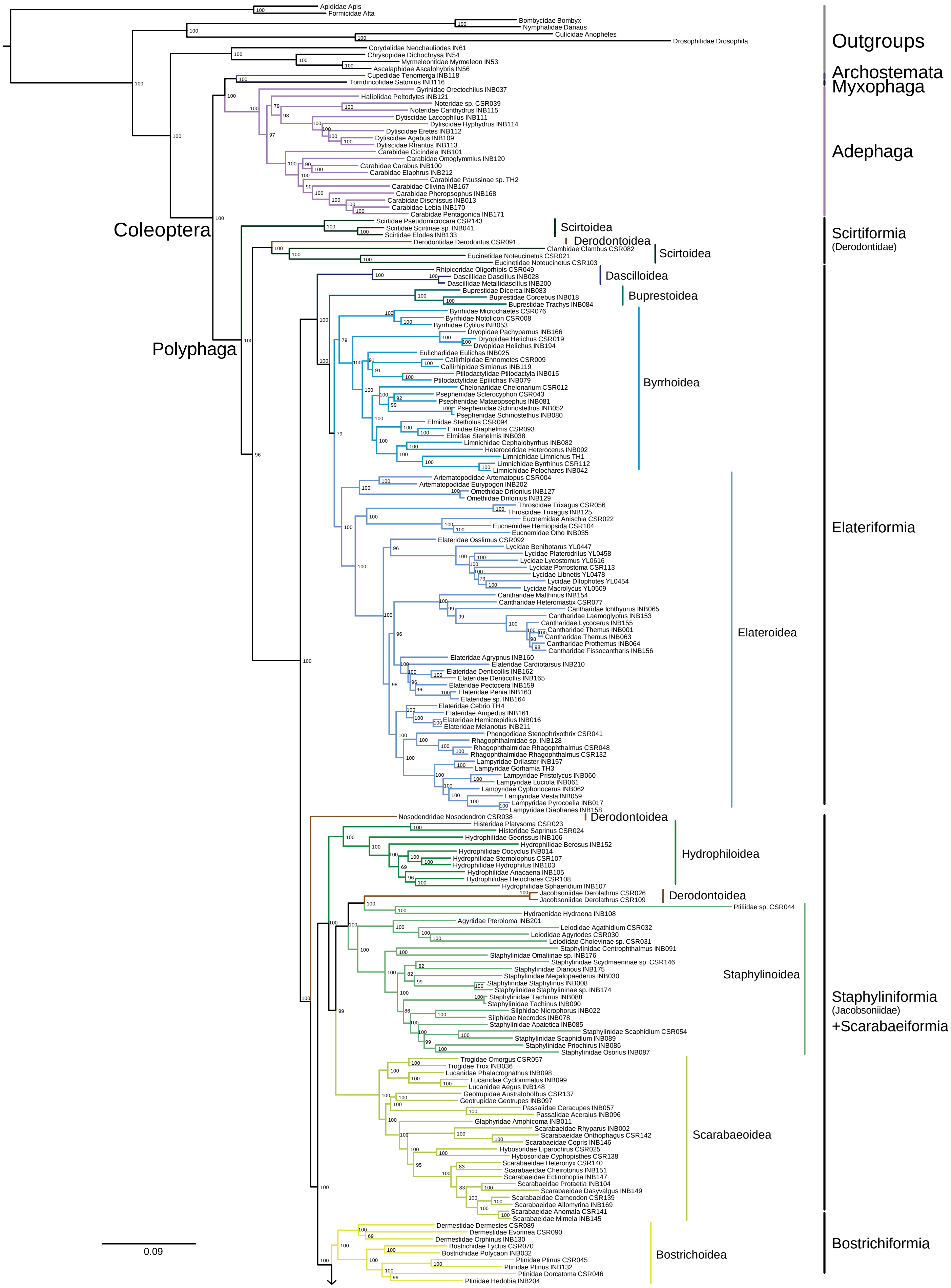

Supplementary Figure 4. The best ML tree inferred by IQ-TREE based on the concatenated degenerated nucleotide matrix (71.406 bp) of 383 species with three codon partitions under the GTR+GAMMA model. Numbers beside nodes are supports values after 5000 ultrafast bootstrap replicates. Different clades are colored according to the classification.

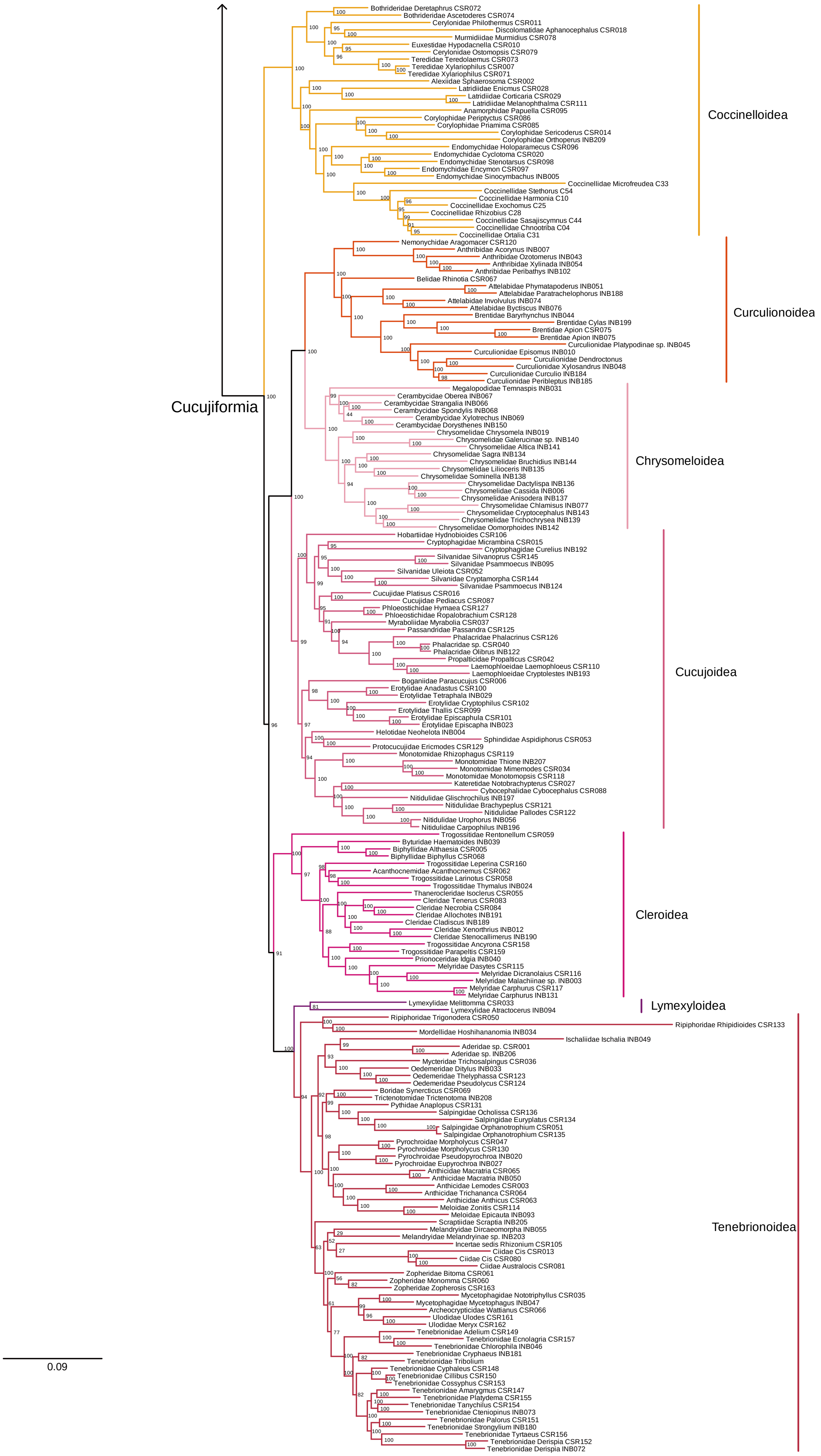

Supplementary Figure 4. (Continued).

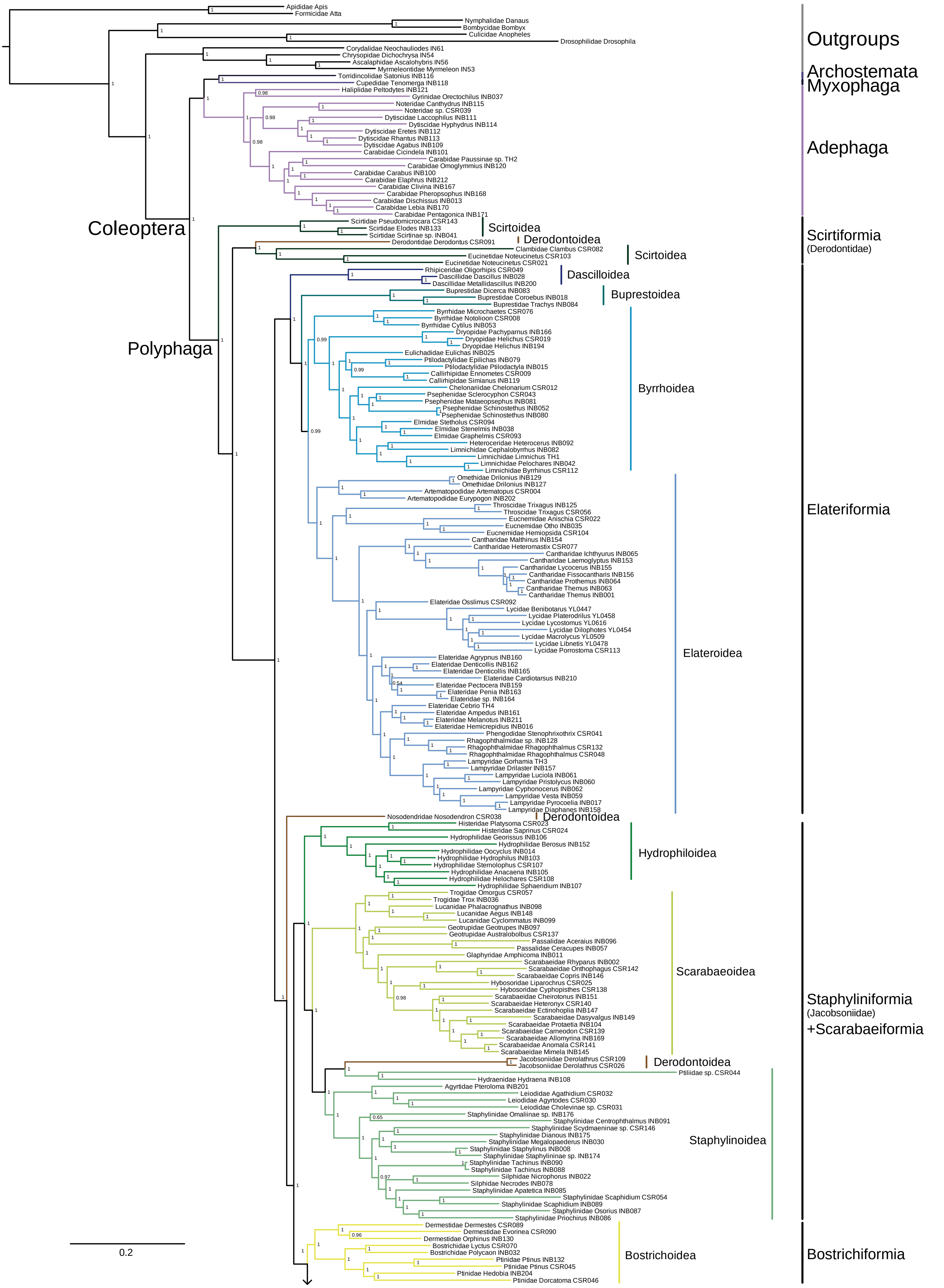

Supplementary Figure 5. Bayesian tree inferred from the concatenated amino acid dataset (23,802 AAs) of 383 species with 10 rate-partitions under their best-fit models. Numbers beside nodes are posterior probabilities. Different clades are colored according to the classification.

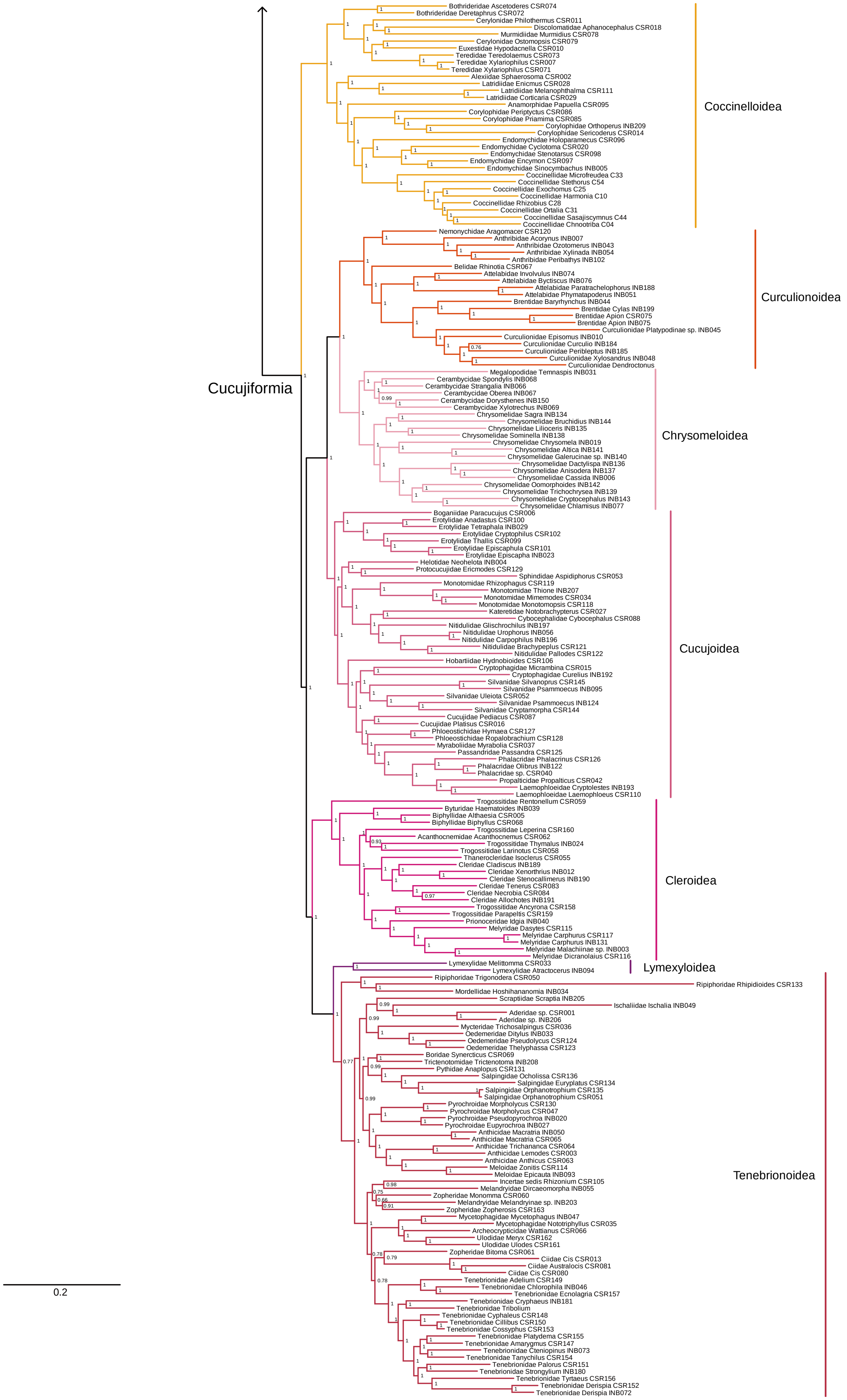

Supplementary Figure 5. (Continued).

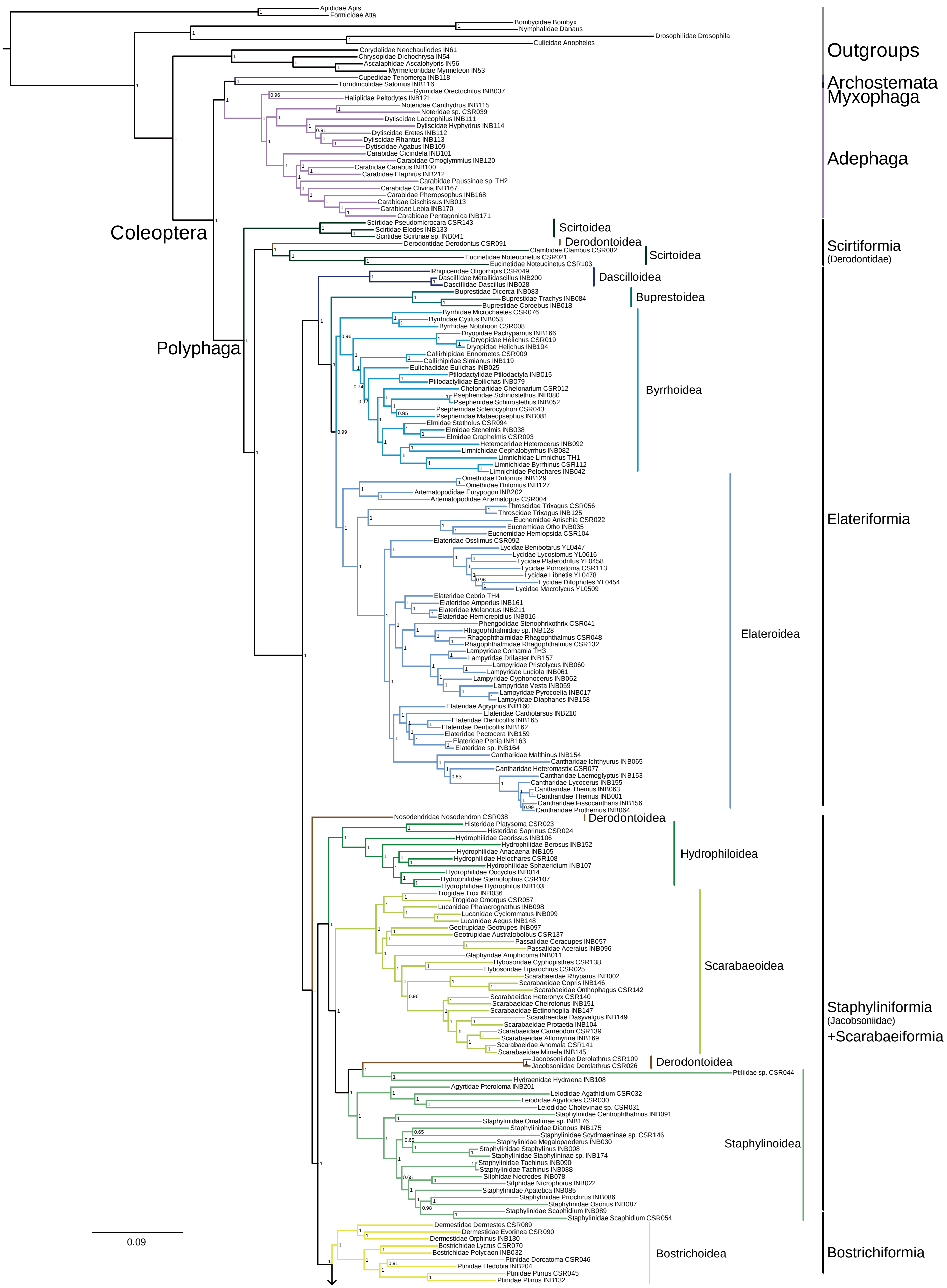

Supplementary Figure 6. Bayesian tree inferred from the concatenated degenerated nucleotide matrix (71,406 bp) of 383 species with three codon partitions under the GTR+GAMMA model. Numbers beside nodes are posterior probabilities. Different clades are colored according to the classification.

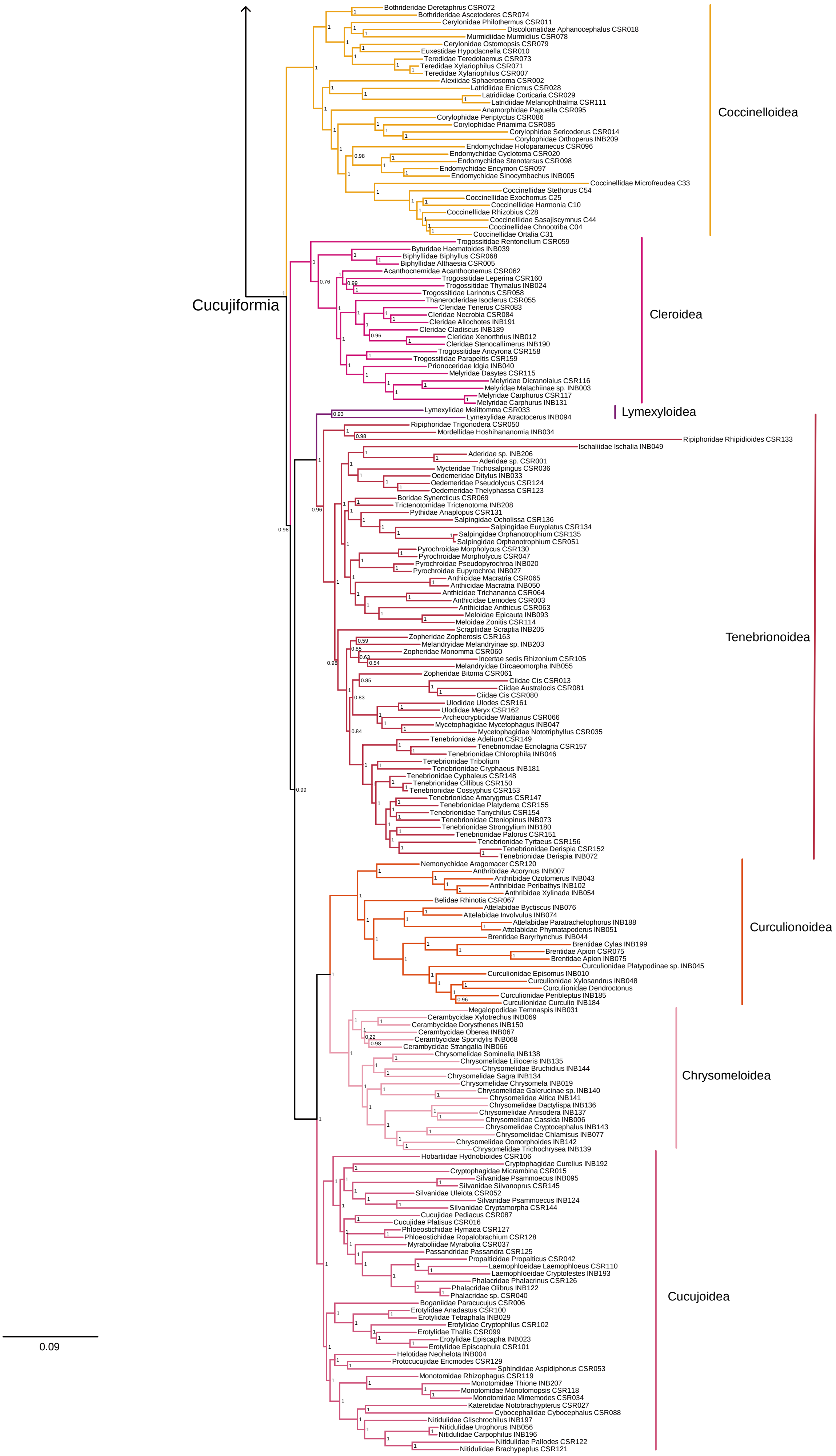

Supplementary Figure 6. (Continued).

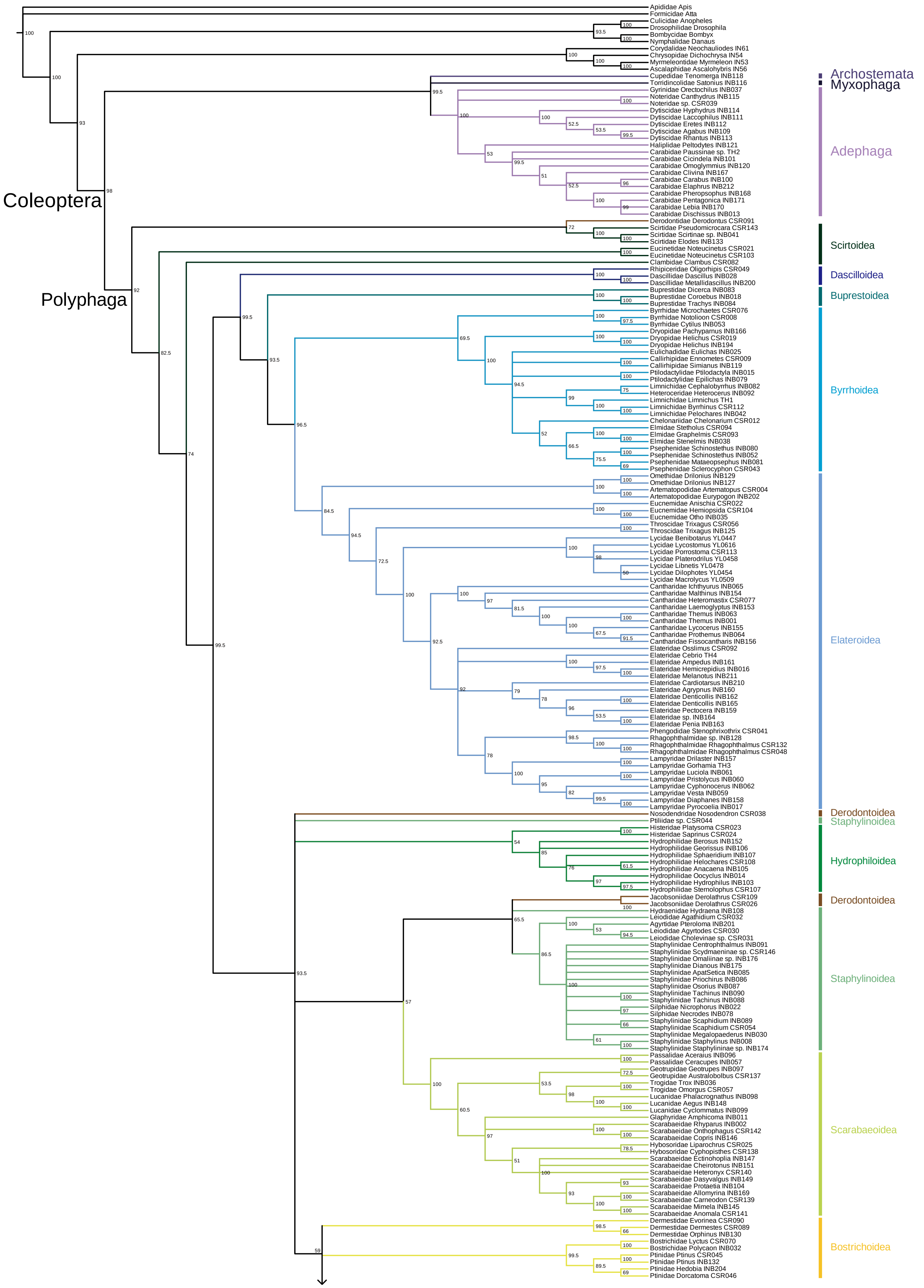

Supplementary Figure 7. Coalescent species tree inferred by ASTRAL from 95 amino acid alignments with low support branches collapsed (<50 bootstrap values). Different clades are colored according to the classification.

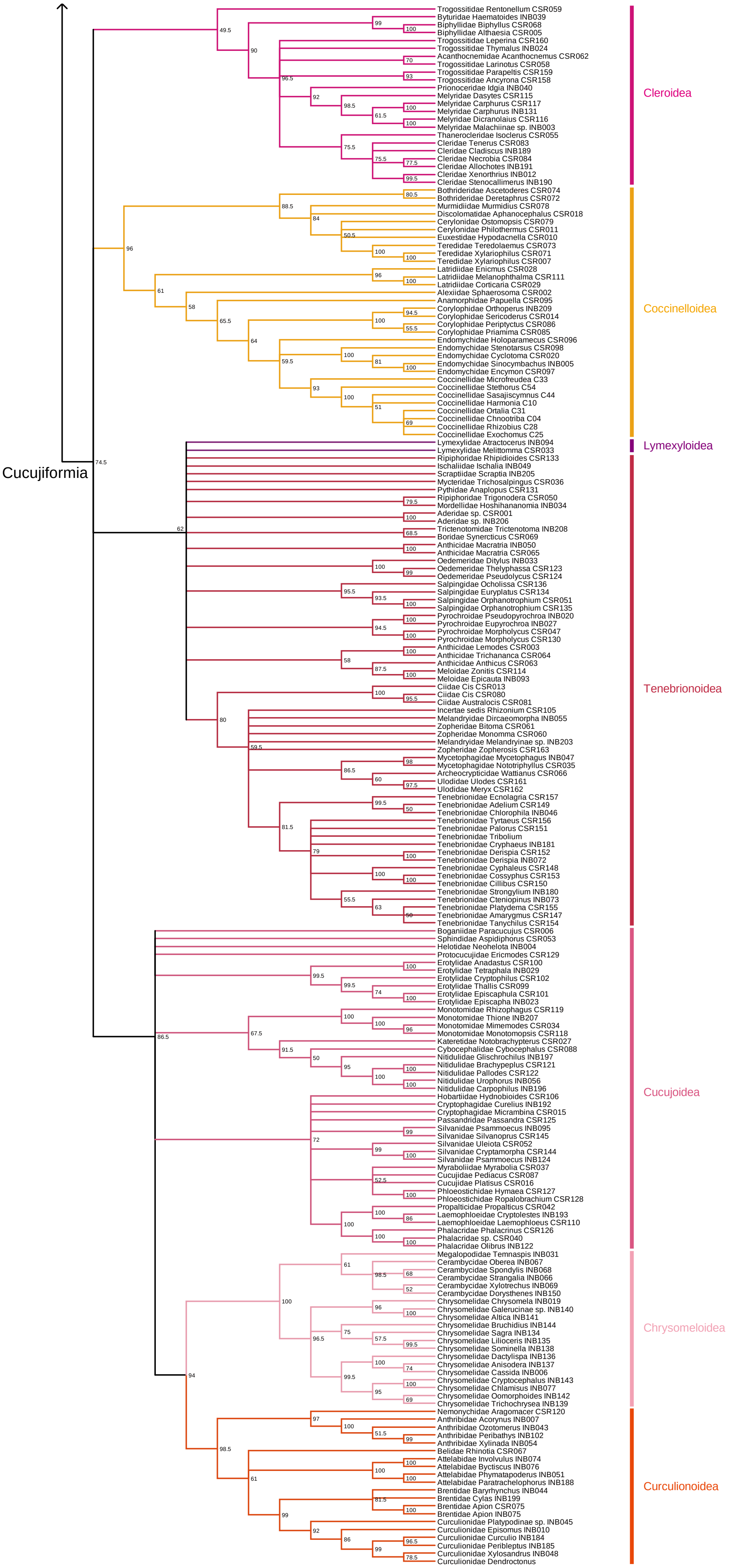

Supplementary Figure 7. (Continued).

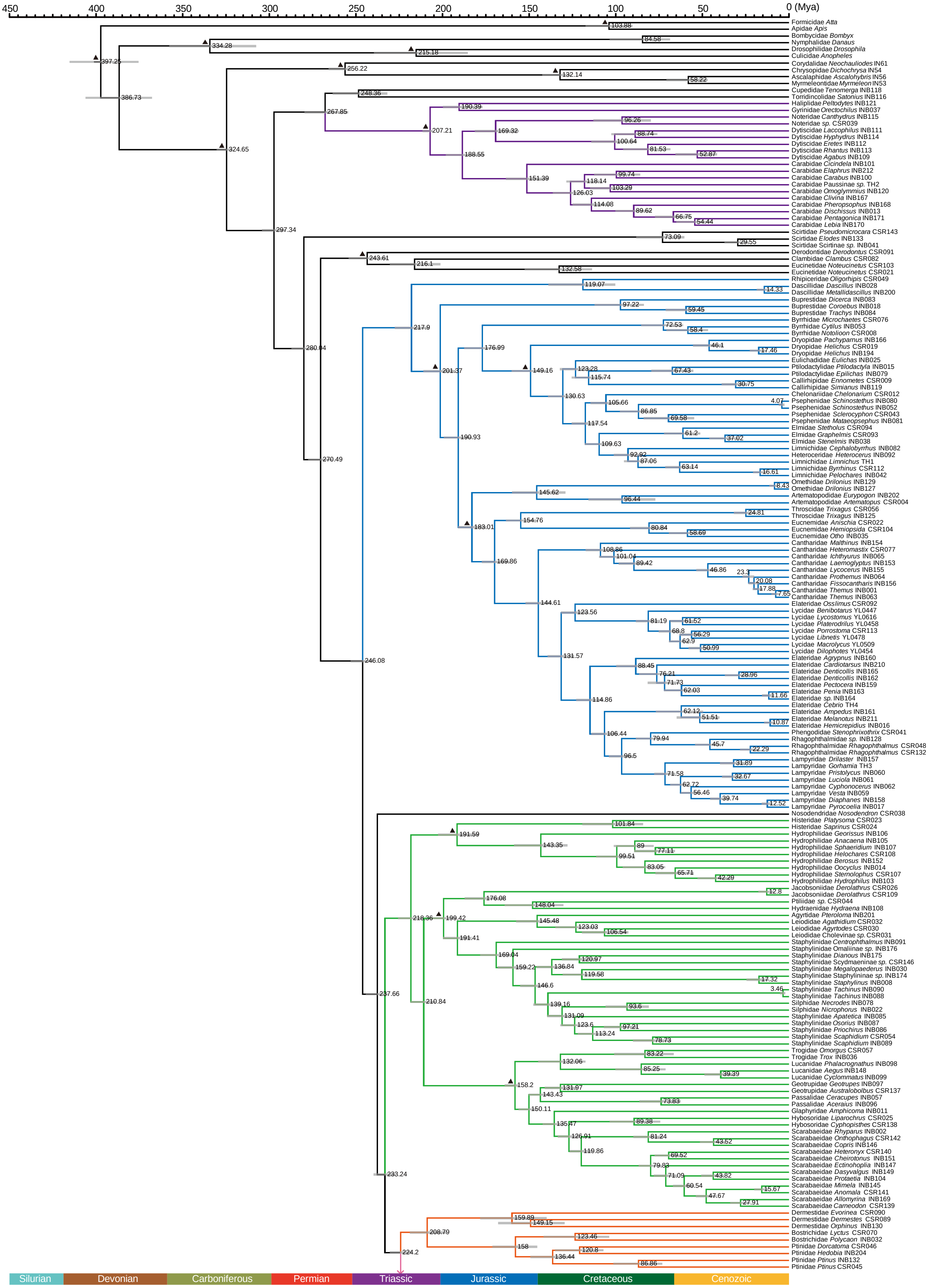

Supplementary Figure 8. Timetree inferred from the amino acid dataset of 383 taxa using fossils at superfamily level. The estimated mean ages are labeled beside nodes. Grey bars represent the 95% credibility intervals of divergence time estimate. Fossil calibration points are shown with black triangles.

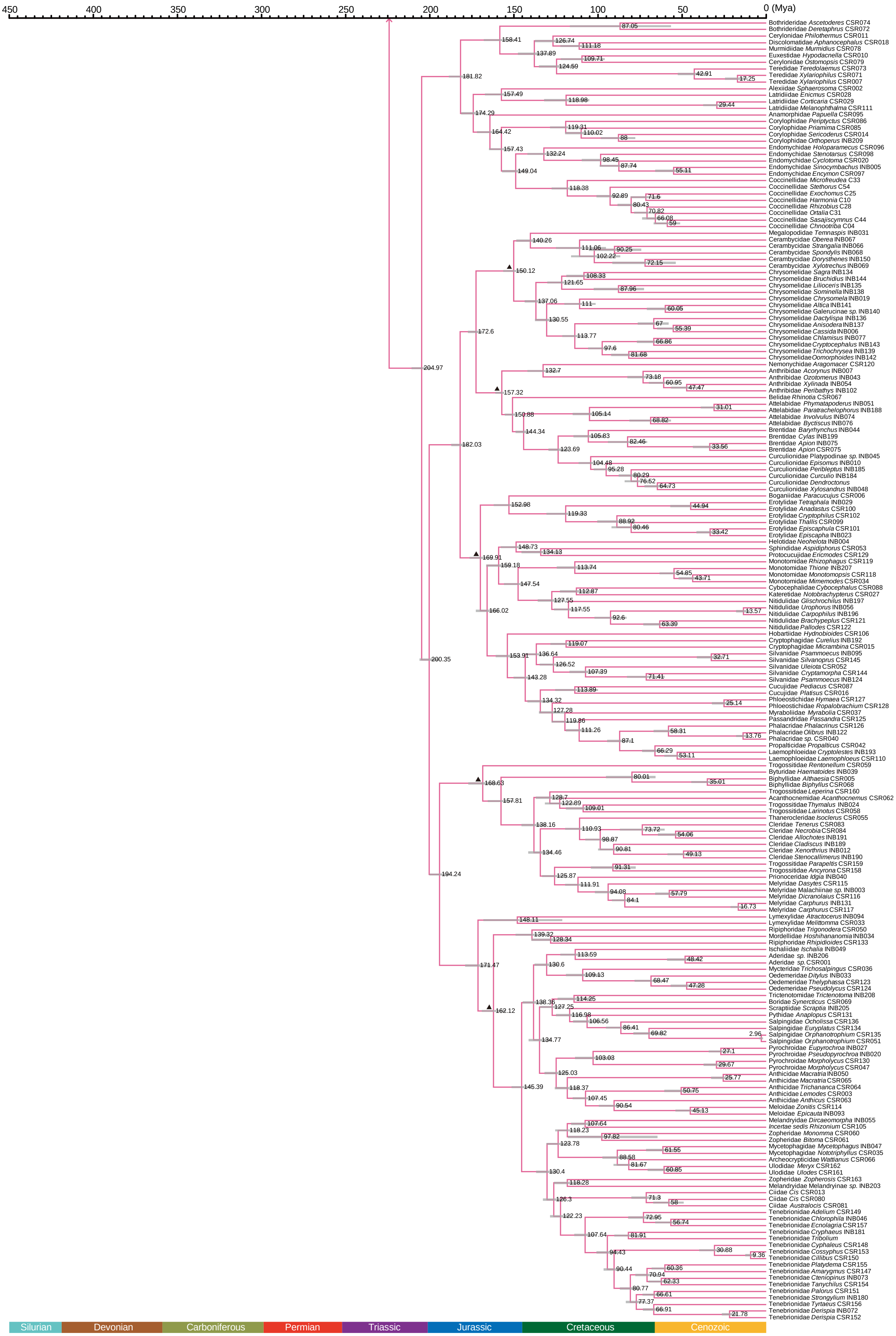

Supplementary Figure 8. (Continued).

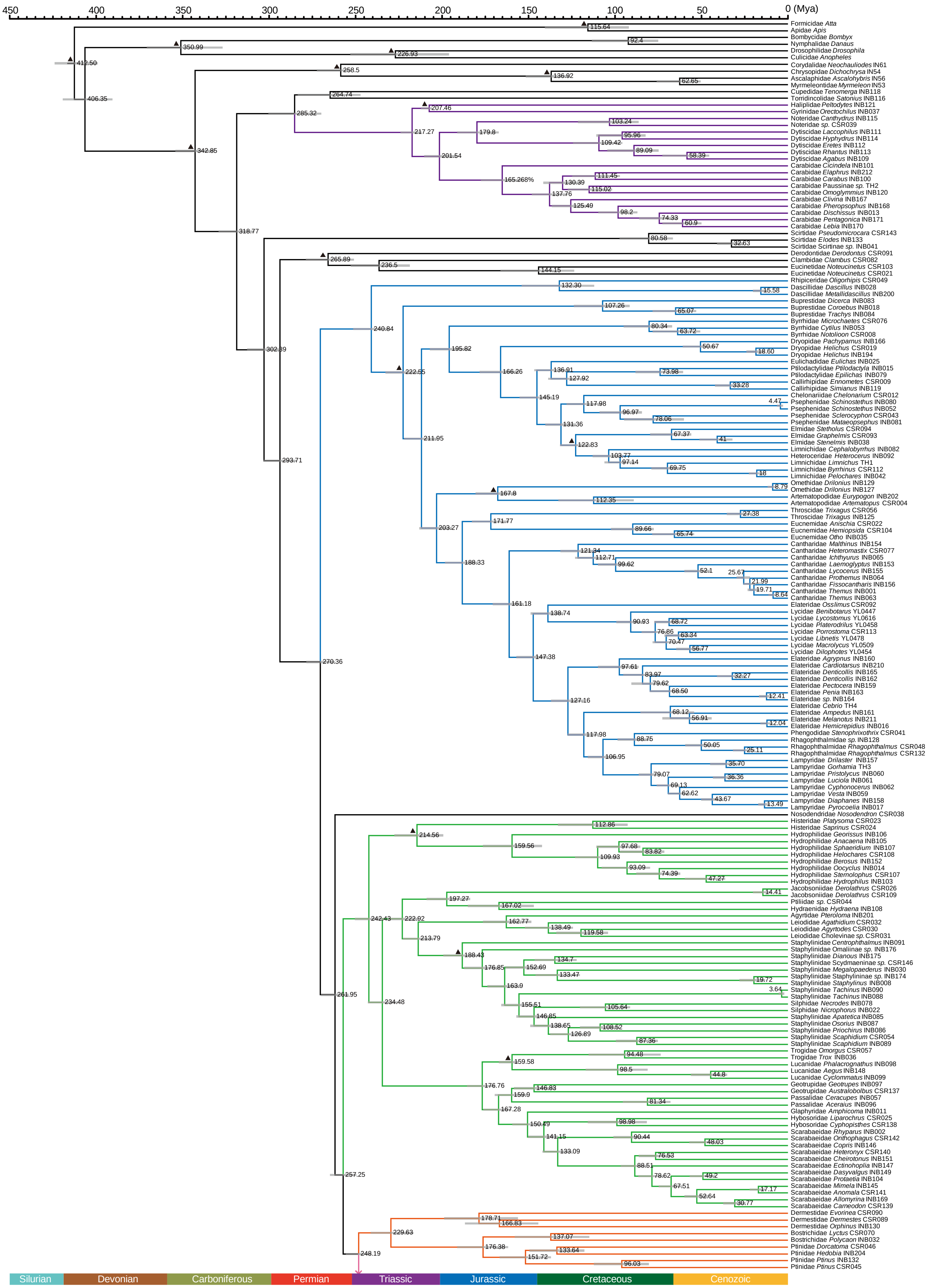

Supplementary Figure 9. Timetree inferred from the amino acid dataset of 383 taxa using fossils at family level. The estimated mean ages are labeled beside nodes. Grey bars represent the 95% credibility intervals of divergence time estimates. Fossil calibration points are shown with black triangles.

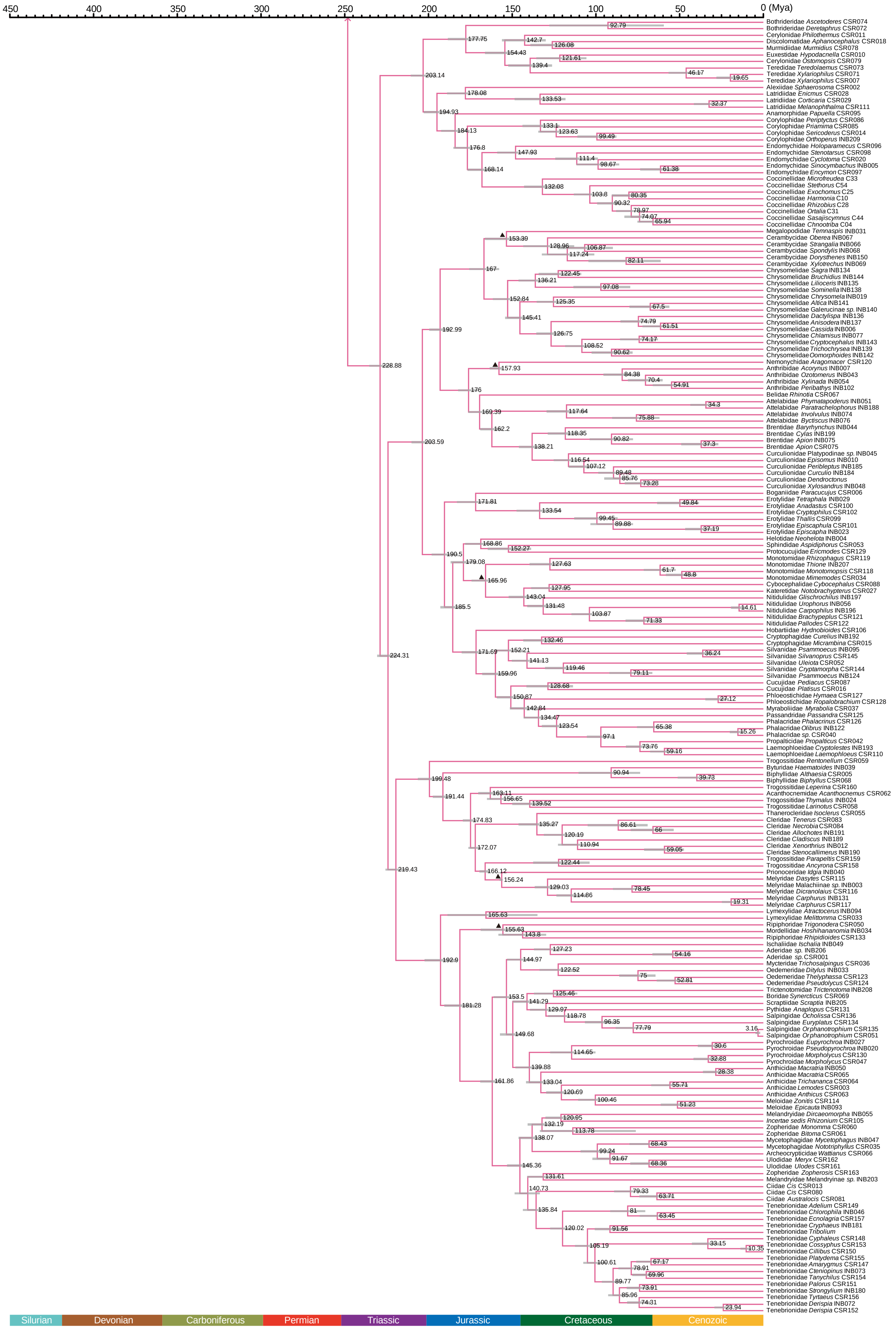

Supplementary Figure 9. (Continued).

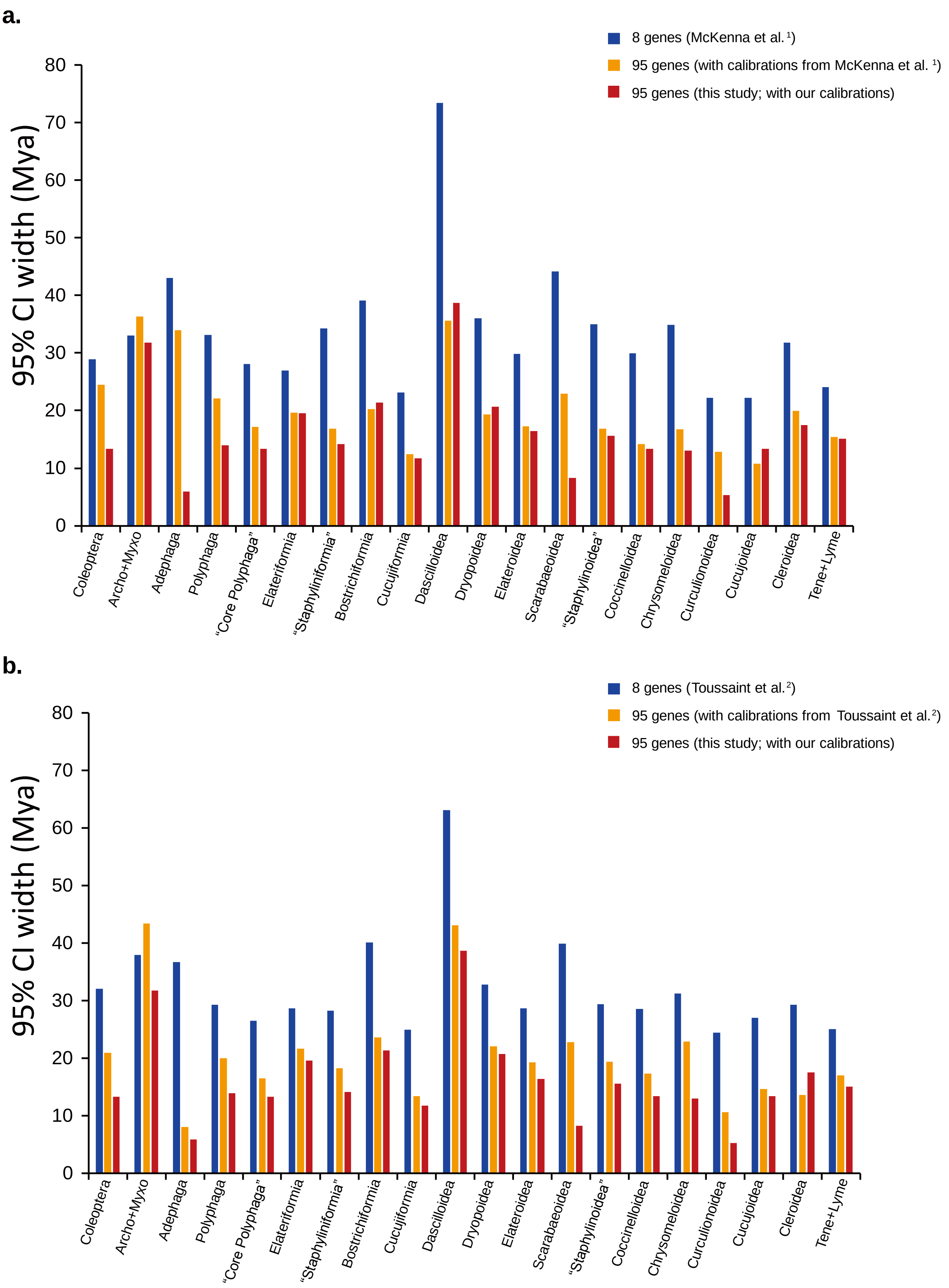

Supplementary Figure 10. Comparison of divergence time confidence intervals of 20 major clades in Coleoptera based on from different datasets and/or calibrations. The blue bars show results from previous studies, the orange ones indicate 95% CIs derived from divergence time analysis using our dataset with others’ calibrations, and the red ones depict results from this study (see Fig. 3; Supplementary Fig. 8; Supplementary Table 2). Clade names correspond to Supplementary Table 2. (a) Divergence times were recalculated using 95 genes with calibrations from McKenna et al.[1]. (b) Divergence times were recalculated using 95 genes with calibrations from Toussaint et al.[2].

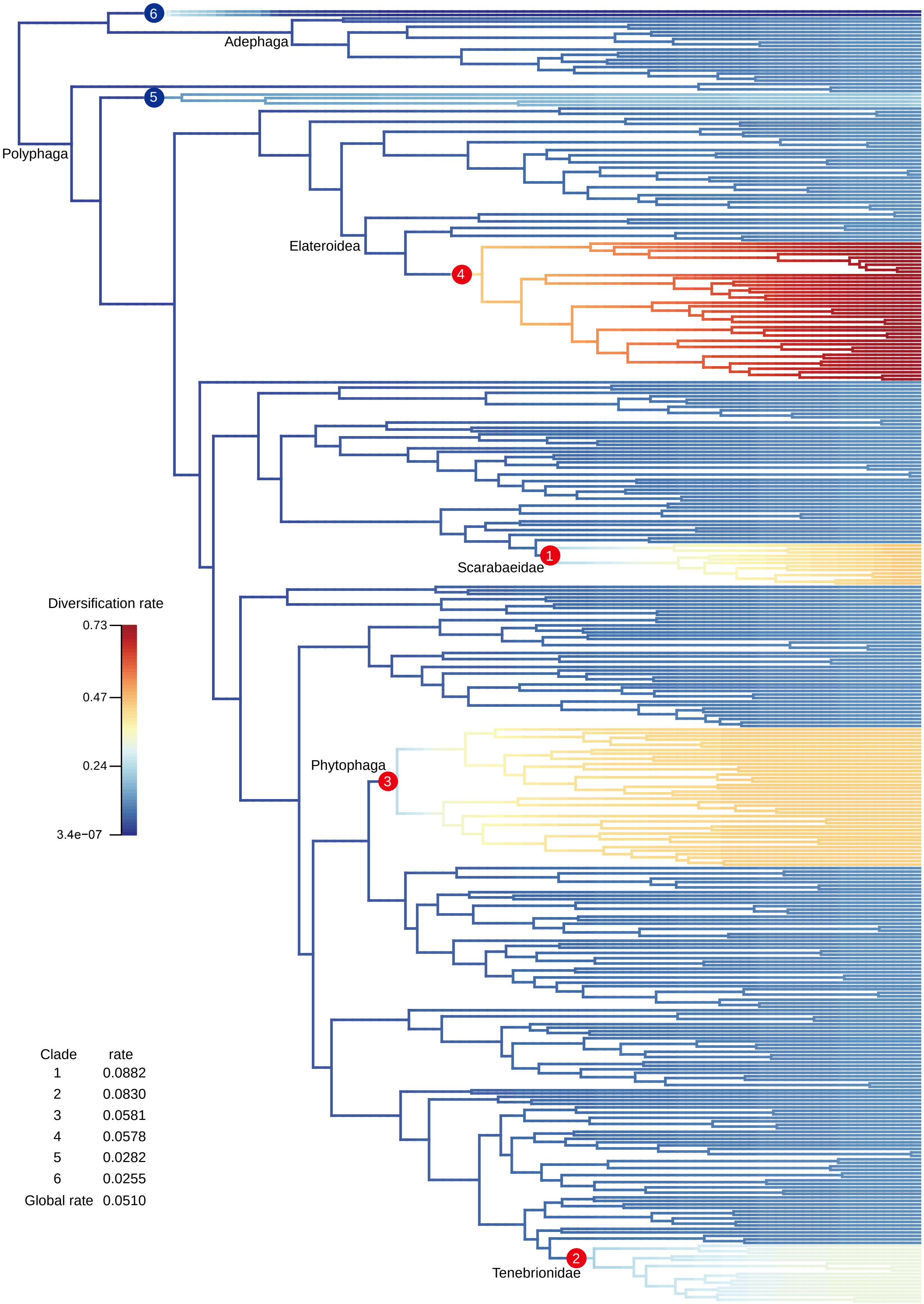

Supplementary Figure 11. The rate shift configuration with the *maximum a posterior* (MAP) probability inferred with BAMM. Branches are colored to represent net diversification rates and warmer colors indicate fast rates. The placements of rate shifts are shown as red circles (rate increases) and blue circles (rate decreases) compared with the global rate. Mean net diversification rates of clades with shifts are included in the lower left.

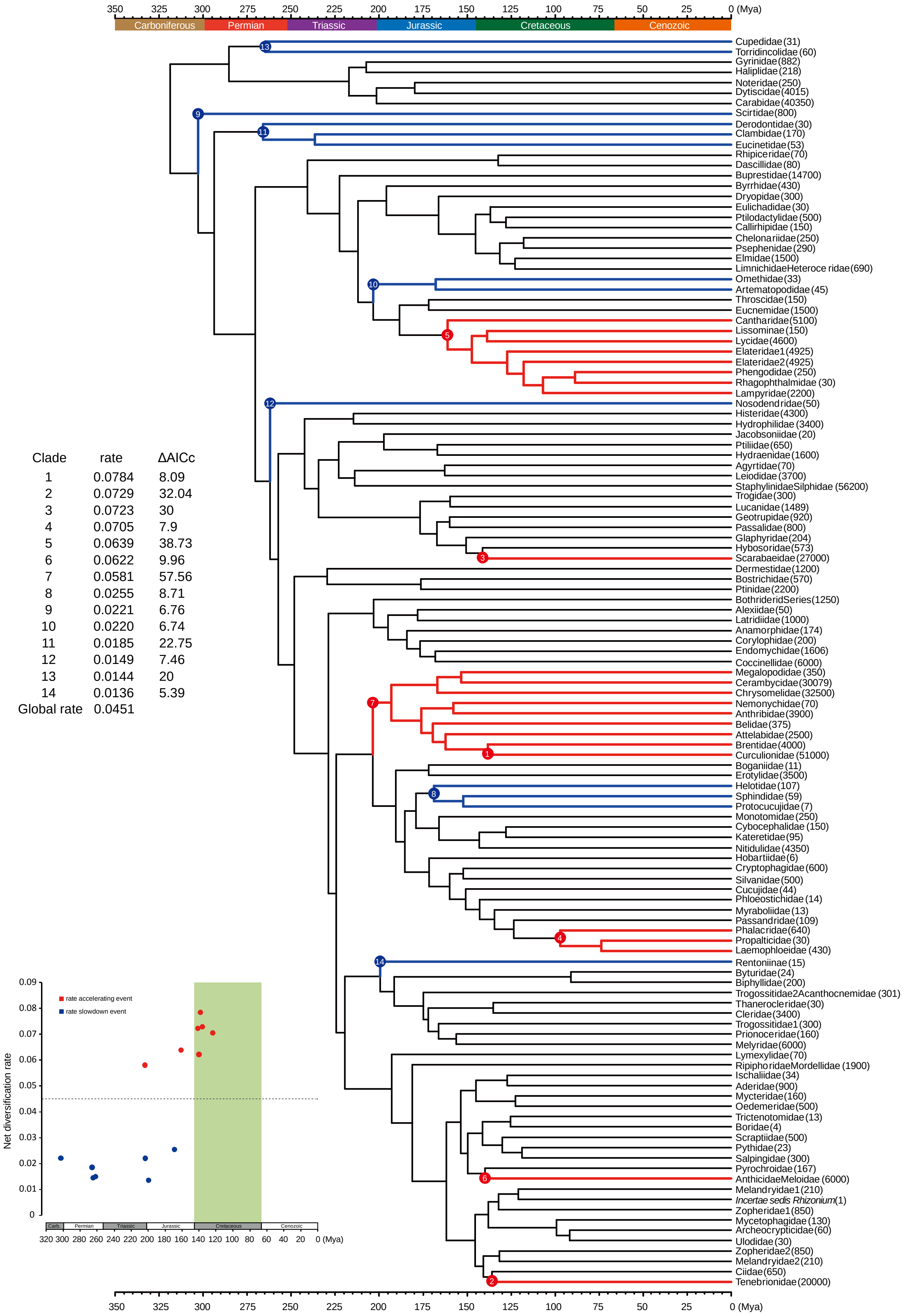

Clade	rate	ΔAICc
1	0.0784	8.09
2	0.0729	32.04
3	0.0723	30
4	0.0705	7.9
5	0.0639	38.73
6	0.0622	9.96
7	0.0581	57.56
8	0.0255	8.71
9	0.0221	6.76
10	0.0220	6.74
11	0.0185	22.75
12	0.0149	7.46
13	0.0144	20
14	0.0136	5.39
Global rate	0.0451	

Supplementary Figure 12. Diversification rate analysis based on the alternative timescale of beetles (see Supplementary Fig. 9) using MEDUSA. Clade with significant diversification rate shifts compared with the background rate are marked with colored branches and circled numbers on the tree (red: rate increase; blue: rate decrease). Timing of the fourteen events is shown in the lower left. Estimated net diversification rates and differences in AICc scores are included in the upper left table.

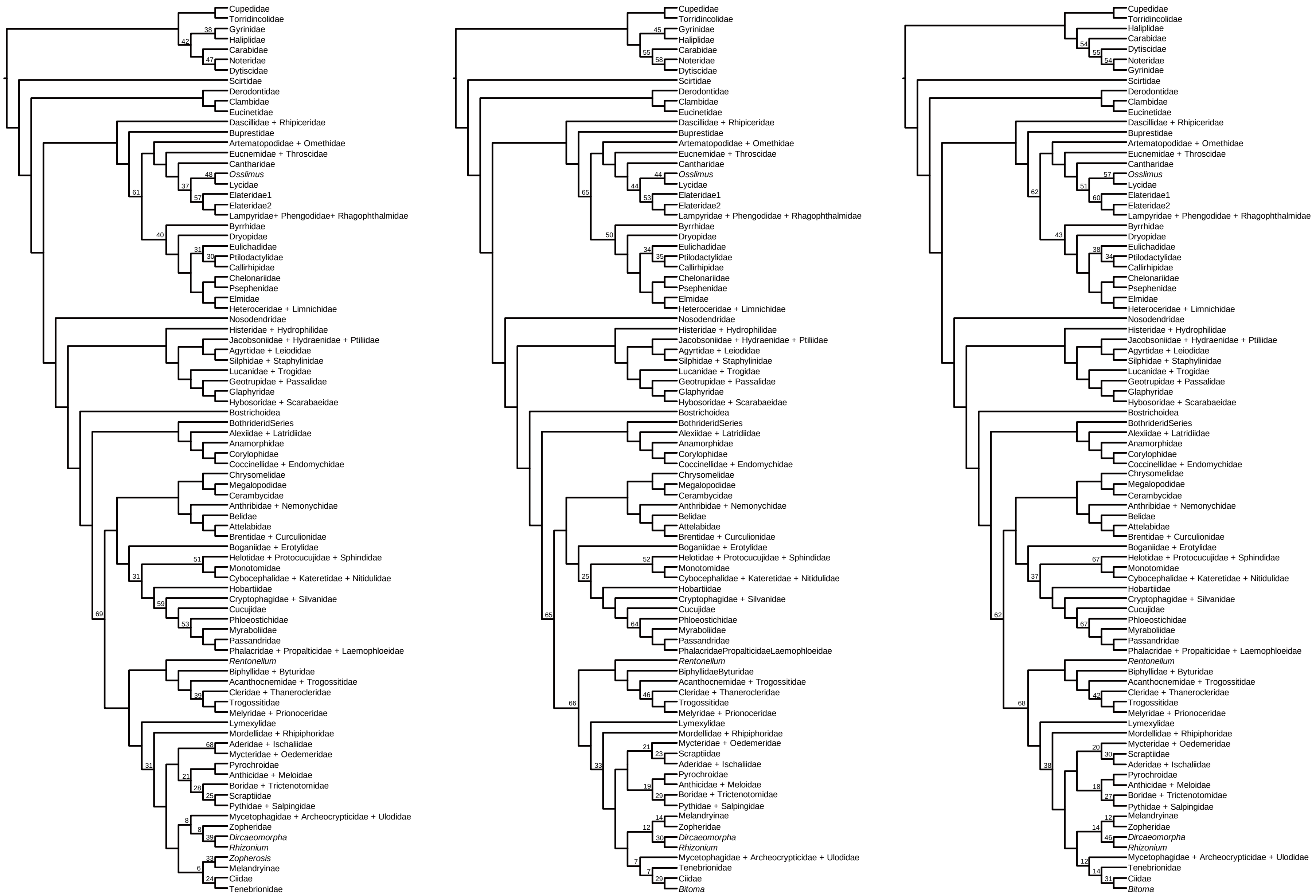

a. 10 rate-binned partitions (with best models)

b. 1 partition (with LG + GAMMA model)

c. 95 gene partitions (with best models)

Supplementary Figure 13. Best ML trees inferred by RAxML based on the concatenated amino acid dataset (23,802 AAs) of 383 species with different partition schemes. (a) 10 rate-binned partitions with best-fit models. (b) unpartitioned with LG + GAMMA model. (c) 95 gene partitions with their best-fit models. The tree topologies show the relationships between families and supra-familial taxonomic groups. Bootstrap values (<70) are shown beside nodes. Note that the partition schemes have little influence to the phylogenetic inference.

Supplementary Table 1. Fossil calibrations used in this study for estimating absolute divergence times.

**Calibrated node**	**Fossils**	**Taxonomy**	**Locality**	**Minimum**	**Maximum**	**References**
Crown Holometabola	*Metabolarva bella*	Holometabola	Piesberg, Lower Saxony, Germany	307.0	-	Nel et al.[3]
MRCA of Formicidae + Apidae	*Kyromyrma neffi*	Formicidae	Sayreville, Middlesex County, New Jersey, USA	89.8	-	Grimaldi and Agosti[4]
MRCA of Lepidoptera + Diptera	*Parasabatinca aftimacrai*	Micropterigidae	Lebanese amber, Lebanon	113.0	-	Whalley[5]
MRCA of Culicidae + Drosophilidae	*Burmaculex antiquus*	Culicidae	Burmese amber, Tanai Village, Kachin, Myanmar	89.8	-	Borkent and Grimaldi[6]
MRCA of Megaloptera + Neuroptera	*Permithone belmontensis*	Neuroptera	Belmont, N.S.W., Australia	252.2	-	Tillyard[7]
MRCA of Chrysopidae + Ascalaphidae	*Lembochrysa miniscula*	Chrysopidae	Chaomidian, Liaoning, China	125	-	Ren and Guo[8]
MRCA of Coleoptera + Neuropterida	*Coleopsis archaica*	Tshekardocoleidae	Grügelborn/Saarland, Germany	295.0	323.2	Kirejtshuk et al.[9]
Crown Adephaga	*Triadogyrus sternalis*	Gyrinidae	Garazhovka, Kharkiv, Ukraine	205.6	-	Ponomarenko[10]
MRCA of Derodontidae + Clambidae	*Juropeltastica sinica*	Derodontidae	Daohugou, Ningcheng, Inner Mongolia, China	155.7	-	Cai et al.[11]
MRCA of Buprestoidea + Byrrhidae	*Ancestrimorpha volgensis*	Buprestidae	Nizhegorodskaya, Russian	164.7	-	Alexeev et al.[12]
Crown Dryopoidea	*Elmadulescens rugosus*	Elmidae	El Soplao amber, Las Peñosas Formation, Spain	109.0	-	Peris at al.[13]
Crown Elateroidea	*Sinobrevipogon jurassicus*	Artematopodidae	Daohugou, Ningcheng County, Inner Mongolia, China	155.7	-	Cai et al.[14]
Crown Hydrophiloidea	*Protochares brevipalpis*	Hydrophilidae	Talbragar Fossil Fish Bed, N.S.W., Australia	150.8	-	Fikáček et al.[15]
Crown Staphylinoidea	Undescribed species	Silphidae	Daohugou, Ningcheng County, Inner Mongolia, China	155.7	-	Cai et al.[16]
Crown Scarabaeoidea	*Juraesalus atavus*	Lucanidae	Daohugou, Ningcheng County, Inner Mongolia, China	155.7	-	Nikolajev et al.[17]
Crown Chrysomeloidea	*Cretoprionus liutiaogouensis*	Cerambycidae	Liutiaogou, Ningcheng County, Inner Mongolia, China	122.5	-	Wang et al.[18]
Crown Curculionoidea	*Eccoptarthrus crassipes*	Nemonychidae	Karatau-Mikhailovka, Kazakhstan	155.7	-	Arnoldi et al.[19]
Crown Cucujoidea	*Jurorhizophagus alienus*	Monotomidae	Daohugou, Ningcheng County, Inner Mongolia, China	155.7	-	Cai et al.[20]
Crown Cleroidea	*Idgiaites jurassicus*	Prionoceridae	Daohugou, Ningcheng County, Inner Mongolia, China	155.7	-	Liu et al.[21]
Crown Tenebrionoidea	*Mirimordella gracilicruralis*	Mordellidae	Huangbanjigou, Liaoning, China	125.5	-	Liu et al.[22]

MRCA = Most recent common ancestor.

Supplementary Table 2. Comparison of divergence times for major nodes shared across four studies.

No.	Crown clade	This study	Toussaint *et al.*[2]	Mckenna *et al.*[1]	Hunt *et al.*[23]
	**Deep nodes**				
1	Coleoptera	297.3(290.8-304.2)	332.9(317.1-349.2)	252.9(238.8-267.7)	285(fixed)
2	Archo + Myxo	248.4(231.7-263.5)	300.4(281.7-319.6)	219.6(204.5-237.5)	227.0(225.3-228.7)
3	Adephaga	207.2(205.6-211.5)	248.3(231.3-268.0)	196.6(174.7-217.8)	237.2(234.6-239.8)
4	Polyphaga	280.0(273.2-287.1)	313.9(299.7-329.0)	229.2(213.5-246.6)	270.5(268.2-272.8)
5	"Core Polyphaga"	246.1(239.8-253.1)	298.1(284.8-311.3)	212.2(199.0-227.1)	249.4*
6	Elateriformia	217.9(208.0-227.5)	266.6(252.8-281.5)	184.6(~171-198)	217.0(206.1-227.9)
7	"Staphyliniformia"	218.4(211.5-225.7)	288.3(274.1-302.3)	200.2(182.8-217.0)	Na
8	Bostrichiformia	208.8(197.5-218.8)	263.5(241.7-281.8)	181.7(161.4-200.5)	219.4(208.2-230.6)
9	Cucujiformia	205.0(199.1-210.8)	274.6(262.3-287.2)	189.8(179.0-202.1)	236.2(228.7-243.7)
	**Shallow nodes**				
10	Dascilloidea	119.1(100.2-138.8)	179.9(149.3-212.4)	120.5(82.4-155.8)	73.1*
11	Dryopoidea	149.2(139.4-160.1)	219.9(203.7-236.5)	146.2(~128-164)	175.9*
12	Elateroidea	183.0(174.7-191.1)	246.0(231.4-260.1)	166.2(151.8-181.6)	188.1(165.9-210.3)
13	"Staphylinoidea"	199.4(192.0-207.6)	280.4(265.5-294.9)	193.2(175.3-210.3)	Na
14	Scarabaeoidea	158.2(155.8-164.0)	221.2(201.9-241.8)	141.1(116.9-161.0)	191.4*
15	Coccinelloidea	181.8(175.4-188.8)	252.6(238.6-267.1)	171.2(157.1-187.0)	202.9(191.5-214.3)
16	Chrysomeloidea	150.1(143.5-156.5)	218.5(203.3-234.6)	145.1(124.6-159.5)	Na
17	Curculionoidea	157.3(155.8-161.1)	226.9(215.3-239.7)	149.6(138.5-160.7)	171.5(144.4-198.6)
18	Cucujoidea	169.9(163.0-176.4)	244.8(231.7-258.7)	167.1(156.2-178.4)	Na
19	"Cleroidea"	168.6(159.7-177.2)	252.1(237.3-266.6)	169.0(152.9-184.7)	190.4*
20	Tene + Lyme	171.5(163.9-179.0)	259.2(246.9-272.0)	175.2(163.7-187.8)	206.5 *

Numbers represent the median times and 95% HPD interval (Mya). Na = Data not available.

"Core Polyphaga" means Polyphaga excluding Scirtidae, Derodontidae, Clambidae and Eucinetidae; "Staphyliniformia" includes “Staphylinoidea”, Hydrophiloidea and Scarabaeoidea;

"Staphylinoidea" includes Jacobsoniidae;

"Cleroidea" means Cleroidea includinging Biphyllidae and Byturidae.

Archo = Archostemata, Myxo = Myxophaga, Tene = Tenebrionoidea, Lyme = Lymexyloidea.

Ages marked with an asterisk were estimated by Mckenna & Farrell[24] from data provided by Hunt *et al.*[23]

Supplementary References

1. McKenna, D. D. *et al.* The beetle tree of life reveals that Coleoptera survived end-Permian mass extinction to diversify during the Cretaceous terrestrial revolution. *Syst. Entomol.* **40,** 835–880 (2015).
2. Toussaint, E. F. A. *et al.* The peril of dating beetles. *Syst. Entomol.* **42,** 1–10 (2017).
3. Nel, A. *et al.* The earliest known holometabolous insects. *Nature* **503,** 257–261 (2013).
4. Grimaldi, D. & Agosti, D. A formicine in New Jersey Cretaceous amber (Hymenoptera: Formicidae) and early evolution of the ants. *Proc. Natl. Acad. Sci. USA* **97,** 13678-13683 (2000).
5. Whalley, P. New taxa of fossil and recent Micropterigidae with a discussion of their evolution and a comment on the evolution of Lepidoptera (Insecta). *Ann. Transvaal Museum* **31,** 71–86 (1978).
6. Borkent, A. & Grimaldi, D. A. The earliest fossil mosquito (Diptera: Culicidae), in Mid-Cretaceous burmese amber. *Ann. Entomol. Soc. Am.* **97,** 882–888 (2004).
7. Tillyard, R. J. Some new Permian insects from Belmont, NSW, in the collection of Mr. John Mitchell. *Proc. Linn. Soc. N.S.W.* **47,** 279-292 (1922).
8. Ren, D. & Guo, Z. G. On the new fossil genera and species of Neuroptera (Insecta) from the Late Jurassic of northeast China. *Acta Zootaxonomica Sinica* **21,** 461-479 (1996).
9. Kirejtshuk, A. G., Poschmann, M., Prokop, J., Garrouste, R. & Nel, A. Evolution of the elytral venation and structural adaptations in the oldest Palaeozoic beetles (Insecta: Coleoptera: Tshekardocoleidae). *J. Syst. Palaeontol.* **12,** 575–600 (2014).
10. Ponomarenko, A. G. Suborder Adephaga, Polyphaga *Incertae Sedis,* Infraorder Staphyliniformia, in Mezozoiskie zhestkokryiye [Mesozoic Coleoptera]. *Akademiya Nauk SSSR, Trudy Paleontologicheskogo Instituta* **161,** 17-119 (1977).
11. Cai, C., Lawrence, J. F., Ślipiński, A. & Huang, D. First fossil tooth-necked fungus beetle (Coleoptera: Derodontidae): *Juropeltastica sinica* gen. n. sp. n. from the Middle Jurassic of China. *Eur. J. Entomol.* **111,** 299–302 (2014).
12. Alexeev, A. Jurassic and Lower Cretaceous Buprestidae (Coleoptera) from Eurasia. *Paleontol. J.* **27,** 9–34 (1993).
13. Peris, D., Maier, C. A., Sánchez-García, A. & Delclòs, X. The oldest known riffle beetle (Coleoptera: Elmidae) from Early Cretaceous Spanish amber. *Comptes Rendus Palevol* **14,** 181–186 (2015).
14. Cai, C. Y., Lawrence, J. F., Ślipiński, A. & Huang, D. Y. Jurassic artematopodid beetles and their implications for the early evolution of Artematopodidae (Coleoptera). *Syst. Entomol.* **40,** 779–788 (2015).
15. Fikáček, M. *et al.* Modern hydrophilid clades present and widespread in the Late Jurassic and Early Cretaceous (Coleoptera: Hydrophiloidea: Hydrophilidae). *Zool. J. Linn. Soc.* **170,** 710–734 (2014).
16. Cai, C. Y. *et al.* Early origin of parental care in Mesozoic carrion beetles. *Proc. Natl. Acad. Sci. USA* **111,** 1–5 (2014).
17. Nikolajev, G. V., Wang, B., Liu, Y. & Zhang, H. Stag beetles from the Mesozoic of inner Mongolia, China (Scarabaeoidea: Lucanidae). *Acta Palaeontol. Sin.* **50,** 41–47 (2011).
18. Wang, B. *et al.* The earliest known longhorn beetle (Cerambycidae: Prioninae) and implications for the early evolution of Chrysomeloidea. *J. Syst. Palaeontol.* **12,** 565–574

(2014).
19. Arnoldi, L. V. Rhynchopora, in Mezozoiskie zhestkokryiye. *Akademiya Nauk SSSR, Trudy Paleontologicheskogo Instituta* **161,** 142-176 (1977).
20. Cai, C. Y., Ślipiński, A. & Huang, D. Y. The oldest root-eating beetle from the Middle Jurassic of China (Coleoptera, Monotomidae). *Alcheringa An Australas. J. Palaeontol.* **39,** 488–493 (2015).
21. Liu, Z. H, Ślipiński, A., Leschen, R. A. B., Ren, D. & Pang, H. The oldest Prionoceridae (Coleoptera: Cleroidea) from the Middle Jurassic of China. *Ann. Zool.* **65,** 41–52 (2015).
22. Liu, M., Lu, W. & Ren, D. A new fossil mordellid (Coleoptera: Tenebrionoidea: Mordellidae) from the Yixian Formation of western Liaoning Province, China. *Zootaxa* **1415,** 49–56 (2007).
23. Hunt, T. *et al.* A comprehensive phylogeny of beetles reveals the evolutionary origins of a superradiation. *Science* **318,** 1913–1916 (2007).
24. McKenna, D. D. & Farrell, B. D. Beetles (Coleoptera). *The Timetree of Life* 278–289 (Oxford Univ. Press, 2009).
