# Supplementary material for: Evolutionary history of Coleoptera revealed by extensive sampling of genes and species
Source: Nat Commun. 2018 Jan 15;9:205. doi: 10.1038/s41467-017-02644-4 (PMC5768713; doi:10.1038/s41467-017-02644-4)
Supplement: Supplementary file 3 — Description of Additional Supplementary Files [file 41467_2017_2644_MOESM3_ESM.pdf]

File Name: Supplementary Data 1
Description: Information of all samples used in this study, including taxonomy, voucher number and collection information. "/"=not applicable, "-"= no information.

File Name: Supplementary Data 2
Description: Information of 95 gene alignments.

File Name: Supplementary Data 3
Description: GenBank accession numbers for newly generated sequences.
